# A Pilot Study of Mindfulness Meditation for Pediatric Chronic Pain

**DOI:** 10.3390/children4050032

**Published:** 2017-04-26

**Authors:** Lynn C. Waelde, Amanda B. Feinstein, Rashmi Bhandari, Anya Griffin, Isabel A. Yoon, Brenda Golianu

**Affiliations:** 1 Pacific Graduate School of Psychology, Palo Alto University, Palo Alto, CA 94304, USA; lwaelde@paloaltou.edu; 2 Department of Psychiatry and Behavioral Sciences, Stanford University School of Medicine, Stanford, CA, 94305, USA; 3 Department of Anesthesia, Stanford University School of Medicine, Stanford, CA 94305, USA; abfein@stanford.edu (A.B.F.); rbhandar@stanford.edu (R.B.); anyag@stanford.edu (A.G.); iayoon@stanford.edu (I.A.Y.)

**Keywords:** mindfulness, meditation, pediatric, chronic pain, adolescent, group therapy

## Abstract

Despite advances in psychological interventions for pediatric chronic pain, there has been little research examining mindfulness meditation for these conditions. This study presents data from a pilot clinical trial of a six-week manualized mindfulness meditation intervention offered to 20 adolescents aged 13–17 years. Measures of pain intensity, functional disability, depression and parent worry about their child’s pain were obtained at baseline and post-treatment. Results indicated no significant changes in pain or depression, however functional disability and frequency of pain functioning complaints improved with small effect sizes. Parents’ worry about child’s pain significantly decreased with a large effect size. Participants rated intervention components positively and most teens suggested that the number of sessions be increased. Three case examples illustrate mindfulness meditation effects and precautions. Mindfulness meditation shows promise as a feasible and acceptable intervention for youth with chronic pain. Future research should optimize intervention components and determine treatment efficacy.

## 1. Introduction

Pediatric chronic pain is a significant problem in the United States, with prevalence rates across pain subtypes ranging from 11% to 38% [1]. Children with chronic pain experience impairments in emotional [2], social [3], and school functioning [4,5]. Chronic pain in childhood not only pervasively impacts quality of life [6], but also leaves youth at risk for progression of pain into adulthood [7]. 

Psychological approaches to pain management are known to be an important part of interdisciplinary treatment for pediatric chronic pain [8]. Such approaches typically include cognitive behavioral therapy [9,10], biofeedback [11], and hypnosis [12]. Research suggests that psychological therapies may improve pain and functioning [13]. 

Mindfulness, often defined as paying attention on purpose and nonjudgmentally in the present moment [14], is a strategy that has received little attention in the field of pediatric pain, despite the growing application of mindfulness-based interventions to address chronic pain in adults [15]. Mindfulness-based interventions for youth have ranged from delivering mindfulness strategies within broader cognitive behavioral packages [16] to developmentally modifying the Mindfulness-Based Stress Reduction (MBSR) program [17]. Jastrowski-Mano et al. [18] published the first small (*n* = 6) randomized controlled pilot study on MBSR for pediatric pain. Due to high attrition and difficulties with recruitment, they were unable to draw conclusions about the efficacy or feasibility of MBSR for this population. Ruskin and colleagues [19] reported on the development of an MBSR group for adolescents with chronic pain and cited challenges as well, including insufficient post-test data which affected their ability to draw conclusions about outcomes. Two more recent studies examining mindfulness-based programs for adolescents with chronic pain indicated that such interventions were feasible and acceptable [20,21]. Chadi et al. [20] utilized a combination of MBSR and Mindfulness-Based Cognitive Therapy adapted to adolescents and reported no improvements in psychological or pain symptoms, though they did report pre/post-reductions in salivary cortisol levels. Ali et al. [21] utilized a developmentally modified MBSR protocol in a pilot cohort study and reported significant pre/post-improvements in fibromyalgia symptoms, anxiety, and functional disability. Given that previous studies utilizing mindfulness for pediatric chronic pain have reported feasibility and some positive outcomes, more research is needed to further explore the acceptability and efficacy of therapeutic modalities that incorporate mindfulness for this challenging patient population. 

The current study was designed to pilot a mindfulness meditation program using a manualized intervention entitled Inner Resources for Teens (IRT) [22], a six-week protocol developed and adapted for teenagers from a similar course utilized for adults named Inner Resources for Stress [23]. Like MBSR, the IRT program teaches mindfulness in daily life and mindfulness meditation, but also includes techniques such as breath-focused imagery and repetition of cue words that are intended to provide additional cognitive structure. Unlike MBSR, IRT does not include hatha yoga or movement-based practices. Previous randomized controlled trials (RCT) of the Inner Resources for Stress intervention for adults have demonstrated pre/post-improvements relative to control groups in diurnal cortisol slope, life satisfaction, and remission from chronic depression [24,25]. 

The aims of this study were to examine the feasibility, acceptability, and usefulness of the mindfulness meditation intervention, as well as the acceptability of specific treatment components of the IRT program. A secondary aim of this study was to document qualitative case descriptions from three participants to highlight benefits and challenges of using mindfulness meditation with this population. 

## 2. Methods

### 2.1. Participants and Setting

Twenty patients diagnosed with chronic pain were recruited from a university-based pediatric pain management clinic. All participants had a history of chronic pain for more than 12 months. They continued to receive multidisciplinary pain management standard of care throughout the intervention. The mean age of participations was 15.1 (standard deviation (SD) = 1.36) and ages ranged from 13 to 17. Two participants (10%) were male. Types of pain included abdominal pain, headaches, fibromyalgia, musculoskeletal pain, complex regional pain syndrome, and joint pain (see Table 1). The mean duration of chronic pain was 2.8 years (range 2.2 to 7). 

### 2.2. Procedures

This study was conducted at a tertiary pediatric pain clinic within a children’s hospital setting on the West Coast in the United States. The study was approved by the university’s Institutional Review Board. Consent and assent procedures took place at the clinic visit. Participants completed baseline questionnaires on the day of the first mindfulness session, including demographics, pain intensity ratings, and domains of physical and psychological functioning. Following the final session, they also completed ratings of pain and function and responded to open-ended questions evaluating the feasibility and acceptability of the intervention. 

### 2.3. Intervention 

The mindfulness intervention consisted of a six-session group-based intervention to teach and practice skills for maintaining sustained nonjudgmental present moment awareness. Three groups were held four months apart, containing nine, three and eight participants, respectively. The intervention was led by a clinical psychologist (author L.W.) and a clinical psychology doctoral student who was trained and supervised in the intervention. The techniques were adapted specifically for adolescents and included a guided body tour, tension release, mindfulness meditation, breath-focused imagery, and repetition of cue words. The Guided Body Tour consisted of noticing and then visualizing breath flow to parts of the body. Complete Breath involved noticing every part of the breath from the inhalation through the exhalation. Repetition of secular cue words consisted of repeating the sounds “Hum Sah,” meant to represent the sound of breathing in synchrony with the breath, repeating “Hum” with every inhalation and “Sah” with the exhalation. Tension Release was a breath-focused visualization exercise designed to enhance participants’ ability to let go of any “tension or holding”.

Sessions were 60 min in length, and began and ended with a 20-min period of therapist-led teaching and practice of mindfulness meditation. The middle 20 min of each session included therapist presentations of didactic material, including the discussion of ways to apply the practice to presenting problems. A major focus of the didactic/discussion sessions was to encourage use of mindfulness skills and techniques in daily life, particularly in response to dealing with pain. Participants were asked to practice these techniques at home for at least 15 min per day for six weeks and to practice them as much as possible in daily life. Participants were provided with a manual, entitled *Inner Resources for Teens* [22] which contained age-appropriate readings and suggestions for the daily practice of the techniques. The manual included four 15-min audio recordings of guided mindfulness meditations that participants used for home practice. The audio recordings were provided on compact disc (CD) and participants were encouraged to convert the recordings to other listening devices. 

### 2.4. Measures

Demographic information collected from participants included age, sex, and duration of chronic pain problem. Qualitative information was obtained from written surveys. 

*Pain Intensity*: Adolescents rated their usual pain intensity and worst pain intensity in the past seven days on the 11-point Numeric Rating Scale (NRS-11) from 0 = “No pain” to 10 = “Worst pain possible” at baseline and post-treatment [26]. The NRS-11 has evidenced validity in the assessment of pain intensity in children and adolescents [26]. 

*Depression:* The Children’s Depression Inventory (CDI) is a self-report assessment of symptoms of depression that includes 27 items that are scored on a 3-point scale [27]. Total scores range from 0 to 54. Psychometric evidence supporting its use in youth are described by other authors [28,29]. 

*Functional Disability:* The 15-item Functional Disability Inventory (FDI) [30] evaluates the physical impact of chronic condition as reported by youth and their caregivers (proxy report) on activities of daily living (e.g., walking, school, sleep) on a Likert scale ranging from 0 = “No trouble” to 4 = “Impossible”, where higher scores signify greater functional impairment. The measure has shown sound reliability and validity when used with youth who experience chronic medical challenges [31].

*The Stanford Pediatric Pain Functioning Inventory* (*SPPFI*): The SPPFI is a clinical tool which evaluates the frequency of pain functioning complaints during the past week. The survey consisted of 24 questions designed for teenagers (aged 13–18) and a similar version for their parents (proxy report). The item stem for the first five items states: “Due to my pain in the past week, it was hard for me to….” Some of the items for this stem include “Walk more than a block,” and “Do physical education (PE), sports, or exercise.” The next 15 items have the stem “Because of my pain in the past week, I….” Some of the items following this stem include “Spent time on my bed or couch,” “Had low energy,” “Had trouble paying attention,” and “Worried about making my pain worse.” The last four items had the stem “In the past week my pain….” Some of the items for this stem included “Interrupted family activities,” “Made my parents worry,” and “Made my parents miss work.” Parent items had the stem “This past week, my child’s pain…” which included items such as “Caused me to worry,” “Affected our family’s activities,” “Caused me/my spouse to miss work.” Participants rated items on a 5-point response scale with 0 = “Never,” 1 = “Almost never,” 2 = “Sometimes,” 3 = “Often,” 4 = “Always”. 

The Project Evaluation questionnaire was administered at post-treatment and included open-ended questions assessing the feasibility and acceptability of the treatment. Participants and parents indicated the desired length and number of sessions the intervention should include. Participants also rated the components of the intervention on a scale 6-point scale, with 0 = “Not sure,” 1 = “Not at all useful,” 2 = “A little bit useful,” 3 = “Moderately useful,” 4 = “Quite a bit useful,” and 5 = “Extremely useful.” Ratings of 0 and 1 were reported as “Not useful,” and ratings of 2–5 were reported as “Useful.” 

## 3. Results

Of the 20 participants enrolled the study, four did not meet the attendance criteria of four out of six sessions (attrition rate = 20%). Attrition was due to transportation difficulties (*n* = 1), a new medical condition that precluded group participation (*n* = 1), and participant concerns that the sessions were not adequately reducing pain (*n* = 2). Two additional participants were lost to follow-up because they were not able to attend the last session and scheduling difficulties prevented them from scheduling an in-person post-treatment assessment; therefore, follow-up assessment data were available for 14 participants. 

Paired *t*-tests examined changes from baseline to post-treatment (see Table 2). Participant ratings of pain intensity (usual pain and worst pain) did not change from baseline to post-treatment. Likewise, participant ratings of depression were unchanged. Although improvements in ratings of functional disability frequency of pain functioning complaints were not statistically significant, there were numerical pre/post-decreases equivalent to a small Cohen’s *d* effect size [32]. Parents’ ratings of the worry caused by their child’s pain significantly decreased from baseline to post-treatment, with a large effect size. 

The program evaluation questionnaire assessed the acceptability of aspects of the intervention. Participants and parents also provided data on the acceptability of the number of sessions the intervention should include (see Table 3). None of the participants indicated that they thought the intervention should be shorter. Less than one-third (*n* = 4; 28.6%) of participants indicated that six weeks was the ideal length for the mindfulness meditation program, and the remaining participants (*n* = 10; 71.4%) endorsed that the intervention should include more than 8 or 10 sessions. None of the participants indicated that the intervention should be shorter than 60 min. The majority of the participants (*n* = 10; 71.4%) indicated that 60 min was the ideal session length and less than a third (*n* = 4; 28.6%) indicated that it should be longer. Only one parent thought the intervention should be shorter in terms of number and length of sessions, and around half of parents agreed that the current format of six 60 min sessions was preferred. Results indicated that the majority of participants rated the intervention components as useful (see Table 4). Practice in daily life and the group sessions were the most highly rated components, with 12 participants (85.7%) rating them as useful. Meditation and repetition of cue words, unguided by an audio recording, was rated as useful by 11 participants (78.6%). Of the four audio recordings of guided practice, Complete Breath and “Hum Sah” were the most highly rated, followed by the Guided Body Tour and Tension Release. 

### Case Illustrations

Case #1. A 15-year-old male participant with multiple pain complaints, including headaches and myofascial pain, was able to learn and practice the meditation techniques and use them to cope with stressors associated with his medical conditions. Although his awareness of pain was slightly increased during the initial sessions, after approximately three weeks of practicing the techniques he became aware that the pain increased his distress and that he could use mindful awareness to reduce his experience of stress, feel more relaxed, and attenuate his experience of pain. He attended all six sessions. He continued to improve after the conclusion of the intervention, by self-report and clinical assessment in follow-up medical visits. 

Case #2. A 14-year-old female with fibromyalgia and depression reported improvements in both physical and emotional symptoms after participating in the full six-week program. She reported that this intervention was helpful and described through the program evaluation questionnaires that she learned a new method for coping with her symptoms. Peer support seemed to be of particular benefit to this participant, as she was able to normalize the experience of chronic pain. She especially liked the practice of cue word repetition during periods of sitting meditation and in her daily life, as she reportedly found that it decreased her worry and rumination and reduced her experience of pain. At the midpoint of the intervention, she reported that mindfulness allowed her to address the emotional impact of chronic pain, particularly anxiety, resulting in self-reported reduction in usual pain level. This improvement increased her ability to begin and maintain a daily exercise program and led to full symptom resolution three months after completion of the intervention. 

Case #3. A 16-year-old female participant with functional abdominal pain and a longstanding history of participation in pain management treatments (e.g., interventional blocks, multiple medication trials and psychological interventions) reported that she utilized distraction as her primary method of coping with chronic pain. Although she reported successful use of the techniques for coping with anxiety, such as when trying to fall asleep or prior to a music competition, she found the concurrent use of mindfulness and distraction to cope with pain to be confusing and ineffective, resulting in exacerbation of pain symptoms. Due to the focus on body awareness with mindfulness practice, she reported that she became more aware of pain sensations and increasingly distressed. With support of the study team, she was withdrawn from the mindfulness program after two sessions. She continued to participate in outpatient pain management, including psychological services. There were no long-term sequelae and her pain level returned to baseline upon termination of the intervention. 

## 4. Discussion

The current study examined a novel intervention for training in mindfulness meditation, Inner Resources for Teens, which combined mindfulness in daily life, meditation practices, breath-focused cue word repetition, and visualization for adolescents with chronic pain. The intervention was reported as feasible, acceptable, and useful for the majority of participants by adolescent- and parent-report (as well as case study description). There were small quantitative improvements in functioning and reduced disability, suggesting a small effect size; however, due to the small number of patients these were not found to be statistically significant. Parent’s concern and worry about their child demonstrated a statistically significant decrease with a large effect size. The adolescents in our pilot study demonstrated acceptable adherence to the intervention, with 80% attending at least four of the six sessions. The majority of participants reported satisfaction with the intervention and expressed the desire for additional sessions after the six weekly sessions were completed. The 60-min session length used in the current study was preferred by most participants and parents. 

The group format of the intervention was a highly rated component, consistent with previous work indicating that social support is a valued component of mindfulness interventions for pediatric chronic pain [21]. Practice in daily life was also highly rated, similar to work with adults showing that integrating mindfulness practice in daily life was more commonly practiced than sitting meditation [25]. At the end of the six-week program, participants preferred the unguided meditation and cue word repetition over practice using audio recordings. It is possible that the guided practices were more important at an earlier stage, when participants were first learning the practices. Of the four audio recordings of guided practice, Complete Breath and “Hum Sah” were the most highly rated, followed by the Guided Body Tour and Tension Release. Both the Guided Body Tour and Tension Release may serve to focus attention on bodily sensations to a greater degree than breath-focused meditations and may be less tolerable for adolescents with chronic pain. 

As illustrated in Case #3, increased body awareness may be difficult for adolescents with chronic pain, particularly if they primarily rely upon distraction as a pain coping strategy. It is notable that all of the intervention components were rated as useful by a majority of participants, suggesting that the combination of mindfulness, cue word repetition, and meditation exercises was acceptable within the scope of a single intervention. This combination of components differs from interventions such as MBSR, which do not include visualization and cue word repetition [17]. However, as illustrated in Cases #1 and #2, these practices were useful for stress and anxiety. Case #1 noted that his pain initially increased as he brought awareness to his breathing and body, but he was able to tolerate the minor exacerbation in pain intensity until he experienced reduced stress and anxiety about the pain, which in turn reduced his perception of pain. Case #2 reported that the use of breath-focused cue word repetition was helpful in redirecting her attention from worry and rumination and provided useful structure during the initial phases of learning the techniques. 

There were no significant changes post-intervention in pain intensity or depression quantitative measures, which is not a surprising finding. Previous studies of mindfulness [20,21] have not reported reductions in pain as an outcome of the intervention, so it may be that these practices are more specific to the distress and dysregulation that accompany the pain experience [33]. Alternatively, it may be that the timing of teaching intervention components is the critical factor. Previous work shows that initial instructions to observe and accept experience—a defining feature of most mindfulness approaches—reduces pain tolerance relative to a control condition and may tax self-regulatory capacity [34]. Longer training may be required to use mindfulness techniques to control or down-regulate the pain experience [35]. Focused practices, such as visualization or cue word repetition, may provide useful structure for teens who are unable to tolerate increased present moment awareness during the initial phase of training. 

Parents demonstrated reduced worry about their child’s chronic pain over the six-week intervention period. It may be that parents had increased confidence in their child’s ability to implement self-management skills, and this confidence decreased parental worry and catastrophizing. Increased parental catastrophizing has been associated with increased child disability in pediatric patients with chronic pain [36], therefore this parent outcome is a promising target for future implementations of mindfulness training. 

Limitations of the study include reliance on self-report measures with only limited inclusion of parent-reported data. In addition, the measure of parent worry was based on an item from an assessment tool (i.e., SPPFI), limiting the interpretation of this construct. Although the heterogeneity of pain complaints may be a limitation that restricts recommending this intervention to specific pain populations, it is also a strength with regard to generalizability across pain conditions in terms of the usefulness of this intervention. Not providing intervention components to family members was an additional limitation that was expressly desired by families and should be addressed in future research.

### Future Directions

Given that most of the teen participants indicated that the number of sessions should be increased, future implementations should consider a longer format (8 to 10 weekly sessions). The 60-min session length used in the current study seemed optimal to most participants. The impact of the intervention on parents’ worry about their child’s pain appears indicative of benefits that parents, themselves, may receive through their child’s knowledge and implementation of pain coping skills. With additional parent-proxy report measures utilized in future studies, it may be possible to further delineate parental perspectives on functional improvements gained via mindfulness interventions, in the context of other parental self-reported outcomes. Future work is recommended to explore how parents may directly benefit from their own mindfulness training within the context of concurrent adolescent and parent groups. Given that previous research and the results of this pilot study indicate that pain experience may initially fluctuate with mindfulness training, introducing more structured practice, such as cue word repetition, during the first session of the intervention may provide the necessary support for patients as they gain skills to reduce anxiety and increase self-regulatory capacity. Likewise, future studies should consider the coping style of individual participants as well as their developmental needs, as the focus of mindfulness practice on present moment awareness and physical sensations may seem overwhelming for some adolescents at first. It may be that initially distraction techniques may provide some much-needed relief from the painful sensations, whereas at a later stage, the use of mindfulness techniques may help to decrease anxiety and amplification of the pain experience. The gradual incorporation of mindfulness techniques into the therapy process may produce optimal results. Additional work is also needed to study the method of delivery as a group intervention compared to individual instruction. The group intervention may allow for much-needed social interaction as well as peer validation, whereas the individualized approach may allow for further personalization of the techniques. Future research may explore the relative merits of both distraction and mindfulness techniques to determine the most beneficial use of these strategies for different pain contexts and strategies. Moreover, improved clarification about the aim and rationale for mindfulness treatment may align participant expectancies with the aims of the intervention during each phase of the training. Because the current brief intervention did not demonstrate decreases in pain or increases in functioning, future studies are needed to explore dose and duration of treatment to optimize pain and symptom management.

## 5. Conclusions

In conclusion, a mindfulness program such as the IRT shows promise as an intervention for helping adolescents with chronic pain and appears to be a feasible tool in the armamentarium of non-pharmacological treatments. The results of this pilot study demonstrate the continued need to further develop pediatric chronic pain mindfulness interventions, exploring a variety of treatment components to determine the most feasible, acceptable and efficacious interventions for this population within the context of a multidisciplinary approach to pediatric chronic pain management. 

## Figures and Tables

**Table 1:** Types of Pain Experienced by Participants.

Pain Type	*n* (%)
Headache	7 (35)
Abdominal pain	6 (30)
Joint pain, rheumatologic	2 (10)
Musculoskeletal pain	2 (10)
Flank pain, polycystic kidney disease	1 (5)
Complex regional pain syndrome	1 (5)
Erythromelalgia	1 (5)

**Table 2:** Means, Standard Deviations, and Effects Sizes of Change from Baseline to Post-treatment.

	Baseline *n* = 20		Post-Treatment *n* = 14			
Variable	*M*	*SD*	*M*	*SD*	*t*	*d*
NRS-usual pain	5.6	1.9	5.6	2.0	−0.4	0
NRS-most severe pain	8.6	1.6	8.8	1.4	−0.8	0
CDI	38.7	7.1	38.9	6.9	−0.1	0
FDI	13.8	6.6	12.5	9.9	0.6	0.20
Teen SPPFI	31.8	14.9	27.4	17.5	0.83	0.30
Parent SPPFI-child’s pain caused me to worry ^a^	2.7	1.2	1.8	1.2	3.3 *	0.75

Note: NRS = Numerical Rating Scale; SPPFI = Stanford Pediatric Pain Functioning Inventory; CDI = Children’s Depression Inventory; FDI = Functional Disability Inventory. ^a^ Parent SPPFI were available for 11 parents. * *p* < 0.01. M = Mean; SD = standard deviation; *t* = paired *t* -test; *d*= Cohen’s *d* effect size

**Table 3:** Participant and Parent Recommendations for Intervention Duration from Project Evaluation.

	Teen Participants *n* (%)	Parents *n* (%)
Number of weekly sessions		
4	0	1 (9.1)
6 (number of pilot sessions)	4 (28.6)	5 (45.5)
8	4 (28.6)	1 (9.1)
10	6 (42.9)	4 (36.4)
Length of weekly session in minutes		
30	0	1 (9.1)
60 (length of pilot sessions)	10 (71.4)	7 (63.6)
90	4 (28.6)	3 (27.3)

**Table 4:** Number and Proportion of Participants Rating Intervention Components as Useful.

Intervention Component	*n* (%)
Inner Resources for Teens manual	10 (71.4)
Guided Body Tour CD	9 (64.3)
Complete Breath CD	10 (71.4)
Hum Sah CD	10 (71.4)
Tension Release CD	8 (57.1)
Meditation practice without CD	11 (78.6)
Hum Sah practice without CD	11 (78.6)
Practice in daily life	12 (85.7)
Group sessions	12 (85.7)

Note: CD = audio recording on compact disc used for guided practice.
